# Disruption of Mitochondrial Homeostasis: The Role of PINK1 in Parkinson’s Disease

**DOI:** 10.3390/cells10113022

**Published:** 2021-11-04

**Authors:** Maria Vizziello, Linda Borellini, Giulia Franco, Gianluca Ardolino

**Affiliations:** 1 IRCCS Foundation Ca’ Granda Ospedale Maggiore Policlinico, Neuroscience Section, Department of Pathophysiology and Transplantation, University of Milan, 20122 Milan, Italy; maria.vizziello@unimi.it (M.V.); giulia.franco@policlinico.mi.it (G.F.); 2 Neuropathophysiology Unit, IRCCS Foundation Ca’ Granda Ospedale Maggiore Policlinico, Via Francesco Sforza, 35, 20122 Milan, Italy; gianluca.ardolino@policlinico.mi.it

**Keywords:** Parkinson’s disease, PINK1, mitophagy, mitochondrial quality control, Parkin

## Abstract

The progressive reduction of the dopaminergic neurons of the substantia nigra is the fundamental process underlying Parkinson’s disease (PD), while the mechanism of susceptibility of this specific neuronal population is largely unclear. Disturbances in mitochondrial function have been recognized as one of the main pathways in sporadic PD since the finding of respiratory chain impairment in animal models of PD. Studies on genetic forms of PD have provided new insight on the role of mitochondrial bioenergetics, homeostasis, and autophagy. PINK1 (PTEN-induced putative kinase 1) gene mutations, although rare, are the second most common cause of recessively inherited early-onset PD, after Parkin gene mutations. Our knowledge of PINK1 and Parkin function has increased dramatically in the last years, with the discovery that a process called mitophagy, which plays a key role in the maintenance of mitochondrial health, is mediated by the PINK1/Parkin pathway. In vitro and in vivo models have been developed, supporting the role of PINK1 in synaptic transmission, particularly affecting dopaminergic neurons. It is of paramount importance to further define the role of PINK1 in mitophagy and mitochondrial homeostasis in PD pathogenesis in order to delineate novel therapeutic targets.

## 1. Introduction

Parkinson’s disease (PD) is the second most common degenerative neurological disorder after Alzheimer’s disease. It is caused by the prominent progressive degeneration of the dopaminergic neurons of the substantia nigra (SN) pars compacta. Molecular mechanisms underlying the degeneration of this specific neuronal population are still largely unclear, but it is known that these cells have a large axonal architecture and a so-called pacemaking activity, which puts them under an extreme bioenergetic demand for the propagation of action potentials, maintenance of membrane potential and synaptic transmission [1,2]. These distinctive features account for the essential role of mitochondrial dysfunction in PD pathogenesis, as demonstrated by the finding that exposure to environmental mitochondrial toxins leads to PD-like pathology [3].

Evidence of this is that PINK1 and Parkin, both involved in mitochondrial dynamics and quality control, are the main cause of autosomal recessive (AR) early-onset PD [4,5,6]. Their molecular role has been extensively studied in the last few years, revealing that the PINK1-Parkin pathway is responsible for the selective degradation of damaged mitochondria, which is necessary for the maintenance of mitochondrial homeostasis, especially in non-dividing cells, such as neurons.

The aim of this paper is to explore the role of PINK-1 in the pathogenesis of PD through a review of the main previously published studies on in vitro and in vivo models. In the end, we will focus on the potential novel therapeutic approaches targeting the mitophagy pathway.

## 2. PINK1 Gene Structure and Most Common Mutations

PINK1 gene mutations are the second most common cause of autosomal recessive early-onset Parkinson’s disease (EOPD) after Parkin (PRKN), representing 1–9% of all genetic PD, both familial or sporadic, varying according to the ethnic population [7], and 15% of all EOPD cases [8].

PINK1 (PTEN-induced putative kinase 1) gene was first outlined by Unoki and Nakamura in 2001 [9], who described its role in the pathogenesis of ovarian cancer. The gene includes eight exonic regions, spans 18 Kb and encodes for a 581 acid serine/threonine-type protein kinase which plays a crucial role in mitochondrial function and metabolism.

In the same year, Valente et al. [10] identified a large Italian Sicilian family with four members affected by autosomal recessive levodopa-responsive early-onset PD. Linkage analysis and haplotype construction revealed a novel locus mapping to chromosome 1p35-p36, which was named PARK6. Later, the description of two additional consanguineous families—respectively coming from central Italy and Spain—further confirmed linkage to PARK6 [11]. Subsequently, multiple candidate genes were identified and sequenced in affected members from each family, revealing the presence of two homozygous mutations in the PINK1 gene, which segregated with the phenotype in the three families [12]: in particular, the Spanish family carried the G309D missense mutation, whereas both Italian families carried a single base mutation in exon 7, which results in the truncation of the C-terminus of the kinase protein.

Several mutations in the PINK1 gene have been reported since its discovery, and most of them are located in the protein kinase domain [13]. Penetrance appears to be complete in individuals who have biallelic PINK1 pathogenic variants.

To date, according to the MDSGene database reports, overall, 151 PINK1 mutation carriers have been described worldwide, with a total of 62 different disease-causing sequence variants involved in both sporadic and familial PD, including 13 definitely pathogenic, 44 probably pathogenic, and 5 possibly pathogenic mutations.

For what concerns the type of mutation, in a systematic review by Kasten et al. [14] among 139 PINK1 mutation carriers-including 116 homozygotes and 23 compound heterozygotes—47.6% were missense mutations, 19.1% were structural variants and 14.3% were nonsense mutations. Most of these mutations reduce allelic expression, causing haploinsufficiency of the protein and impairing mitochondrial function. Other mutations might instead induce gain of toxic function, also leading to mitochondrial dysfunction and neuronal death [15].

The most frequently reported mutation is p.Gln456Stop [16]. As shown by Grunewald et al. [17], this mutation, when in homozygosity, exerts a major effect on mRNA level with an 80–90% reduction, most likely via nonsense-mediated mRNA decay, determining lower levels of *PINK1* transcript.

Considering exclusively index cases, the most common mutation is p.Leu347Pro [14].

Although PINK1-related PD is associated with homozygous or compound heterozygous mutations, different heterozygous mutations have been reported in PD patients and healthy controls [18,19,20]. However, the effect of heterozygous mutations and their potential role as a risk factor for PD is far from being detangled, with some authors reporting a higher prevalence of heterozygous rare variants in patients when compared to controls [18,21].

## 3. PINK1 Protein Functions

PINK1 encodes a 581 acid serine/threonine-type protein kinase localized primarily in mitochondria, where it plays a pivotal role in regulating mitochondrial quality control (*mitoQC*), promoting maintenance of respiring mitochondrial networks, and regulating the selective elimination of damaged mitochondria via autophagy, a process known as mitophagy [22].

Aside from its role in mitochondrial quality control, PINK1 is also known to have a pro-survival role in preventing neuronal cell death in response to various stress conditions [5,23,24].

**Figure 1:**
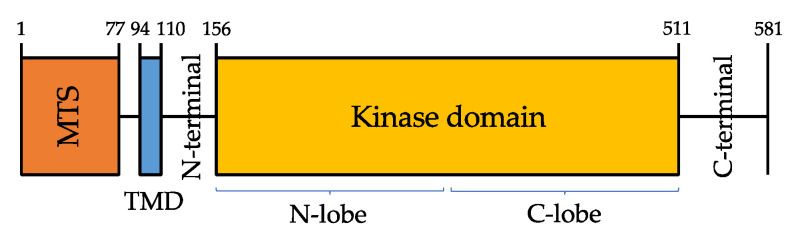
Domain architecture of PINK1 (581 amino acids). Mitochondrial targeting sequence (MTS, orange), transmembrane domain (TMD, blue).

PINK1 protein is composed of a highly conserved Ser/Thr kinase domain flanked by a C-terminal, a transmembrane sequence (TMS, or TMD, transmembrane domain), and an N-terminal mitochondrial targeting sequence (MTS) [25] (Figure 1).

PINK1 kinase domain consists of amino- and carboxy-terminal lobes (N-and C-lobes), but it also has a unique feature, that is the presence of three amino acid sequences—called insertions, in addition to an unusual domain in the C-terminal region (CTR) that is not found in any other protein kinase [26].

Kumar et al. [27] and Schubert et al. [28] in 2017 observed that insertion 2 contains a β-strand and an á-helix which relocate the N-lobe next to the so-called áC helix, a conserved key regulatory region of many protein kinases; this region is likely to play a role in regulating PINK1 enzymatic activity.

Moreover, the CTR and the C lobe share a hydrophobic core which explains why CTR and the kinase domain cannot be drawn apart. For what concerns insertion 3, Schubert et al. [28] described its role in substrate binding: mutations in this insertion impair ubiquitin-like domain (UBL) and ubiquitin phosphorylation, but do not affect PINK1 autophosphorylation.

Limited information is available, instead, about the role of both CTR and insertion 1.

According to both Kumar and Schubert models, most of the disease-causing PINK1 mutations associated with PD may act altering PINK1 or selectively impairing its catalysis, phosphoregulation, or substrate binding [26].

Elucidating the mechanisms that underlie the physiological and pathological PINK1 functioning will provide a rationale for the identification of potential PD-modifying treatments.

Dopaminergic neurons in both PD and aged individuals display dysfunctional mitochondria accumulating high levels of mitochondrial DNA (mtDNA) deletions [29,30].

Mitochondrial function impairment is strongly implicated in the etiology of PD: the first evidence for mitochondrial involvement in PD pathogenesis was the finding that chemical inhibition of mitochondrial Complex I could reproduce Parkinsonism in humans and animals [31,32], because inhibition of this complex induces both depletion of ATP and generation of ROS which causes oxidative stress. Moreover, the administration of the mitochondrial respiratory poison MPTP (1-methyl-4-phenyl-1-1,2,3,6-tetrahydropyridine) in mice induces symptoms similar to those observed in sporadic PD.

All the four principal genes implicated in autosomal recessive EOPD-PRKN, PINK1, DJ-1, and VPS13C—are involved in mitochondrial homeostasis and mitophagy.

In healthy mitochondria, under normal conditions, PINK1 levels are often repressed due to its translocation to the inner mitochondrial membrane (IMM), where its two domains MTS and TMS are cleaved off by, respectively, MPP (mitochondria processing peptidase) [33] and PARL (Presenilin-associated rhomboid-like protein) [34]. When deprived of MTS and TMS, the resulting 52 kDa processed PINK1 is transferred to the cytoplasm and then degraded by the ubiquitin-proteasome system [35]. In response to mitochondrial stress/depolarization, the altered IMM prevents MTS and TMS from being cleaved. Full-length PINK1 is stabilized and accumulates on the mitochondrial outer membrane (OMM) where it forms a multimeric complex with outer-membrane proteins, the TOM machinery, and self-phosphorylates on Ser228, Thr257, and Ser402 to fully activate its kinase domain [36]. Activated PINK1 phosphorylates Parkin at Serine65 (Ser65) in the N-terminal ubiquitin-like (UBL) domain, to activate Parkin enzymatic function and induce Parkin recruitment into the OMM of the damaged mitochondria [37,38]. Accumulation of PINK1 is crucial as a mitochondrial damage sensor, but the molecular mechanisms underlying its association with the TOM complex remain unclear [39].

Moreover, PINK1 is also known to phosphorylate ubiquitin itself at Ser65, which then binds to the RING1 domain of Parkin with high affinity [40,41,42], facilitating its conformational rearrangements and enzymatic activation.

For full activation of Parkin, phosphorylation of both the N-terminal UBL Ser65 residue of Parkin and ubiquitin is required.

Activated Parkin further attaches ubiquitin to neighboring OMM proteins so that PINK1 can recognize them as damaged proteins and phosphorylate them, which in turn amplifies Parkin activation and recruitment [43]. PINK1/Parkin conjugated actions cause damaged mitochondria to be coated with phosphorylated Ser65-ubiquitin chains and headed towards degradation into the proteasome.

Regarding PINK1 regulation, another important role is that of phosphatase PTEN, a de-phosphorylating intracellular enzyme that negatively regulates the activity of many protein kinases. PTEN-L splicing variant, which contains an additional domain in its N-terminal, is generally located in the OMM, where its activation antagonizes PINK1-mediated phosphorylation of Parkin-UBL and ubiquitin, thus blocking mitophagy [44].

PINK1 also coordinates several aspects of mitochondrial quality control, influencing the clearance of a wide range of substrates. Growing evidence suggests that PINK1 and Parkin are crucial for modulating mitochondrial fission and fusion, which balance is critical for the mitochondrial network. Indeed, it is known that ubiquitination of mitofusins (MFN) 1 and 2 is an early event in the PINK1-Parkin-dependent pathway. The MFN are transmembrane GTPases located in the OMM and implicated in the fusion of mitochondria. Their Parkin-dependent ubiquitination and subsequent proteasomal degradation prevent them from promoting damaged mitochondrial refusion [45]. This process further leads to fragmentation of damaged mitochondria [46], thus promoting mitophagy. The first hypothesis about MFN-1/-2 in mitophagy is that PINK1/Parkin-dependent ubiquitination of MFNs could induce the recruitment of ubiquitin-binding proteins which could, in turn, activate the formation of autophagosomes for damaged mitochondria; alternatively, ubiquitination of MFNs could degrade the pro-fusion proteins preventing the refusion of depolarized/damaged mitochondria with the mitochondrial network, thus contributing to segregate damaged mitochondria for degradation by mitophagy [47].

PINK1 deficiency also affects Complex I activity, thus disrupting respiratory chain function and increasing cell susceptibility to apoptosis [48]: Morais et al. [49] described that Complex I phosphorylation mediated by PINK1 is essential for ubiquinone reduction to regulate mitochondrial bioenergetics. The disruption in respiratory chain function results in cellular ATP deficiency and subsequent reduction in mitochondrial membrane potential (ÄØm), which induces Parkin translocation to the OMM, where it ubiquitinates various OMM proteins and promotes the selective removal of damaged mitochondria (mitophagy). Moreover, the use of the uncoupler CCCP (carbonyl cyanide m-chlorophenyl hydrazone) in mammalian cells reduces ÄØm, inducing Parkin translocation to the OMM and damaged mitochondria elimination via mitophagy.

Aside from their role in mitophagy and mitochondrial quality control, both Parkin and PINK1 have independent pro-survival activities and can prevent neuronal cell death in several stress paradigms [50].

In 2012, Soubannier et al. [51] firstly described an additional role of the PINK1/Parkin pathway in the regulation of a selective form of mitochondrial quality control, based on the presence of cargo-selective mitochondria-derived vesicles (MDVs). MDVs bud off mitochondria and are responsible for the degradation of damaged and oxidized mitochondrial proteins and lipid cargo into peroxisomes and other specific cargos into lysosomes, in a way that is independent of autophagy. Interestingly, at least part of MDVs trafficking is mediated by the vacuolar protein sorting 35 homolog gene (VPS35) [52], which is known to be associated with late-onset autosomal dominant PD (PARK17) [53,54].

More recent studies showed the existence of an intimate connection between mitochondria and other organelles, particularly the endosomal compartment [55,56,57].

Even if little is known about this complex interaction, it is likely that it plays a crucial role in influencing many mitochondrial functions, some of them regarding mitochondrial quality control processes and the release of MDVs. In fact, many proteins involved in endocytosis are also part of the process that regulates mitochondrial fission and fusion and, on the other hand, PINK1 and Parkin widely contribute to regulate mitochondrial quality control mediated by the lysosomal-dependent degradation of dysfunctional and damaged mitochondria. In this setting, PINK1-related mitochondrial dysfunction promptly results in the accumulation of altered endosomal components [58], thus suggesting this cross-talk represents an efficient and important way for metabolites to be recycled [59].

Nevertheless, the exact mechanisms through which mitochondria-endosome cross-talking occurs in mammalians is still unclear, as well as its implications in physiological and pathological conditions, thus limiting so far our chance to outline its possible role in PINK1-related mitochondrial impairment.

PINK1 overexpression has also been associated with reduced toxin-mediated cell death, supporting the hypothesis of a pro-survival role of this protein [60,61]. On the other hand, PINK1 KO mice show increased vulnerability to the complex I inhibitor MPTP, which moreover can be rescued by Parkin [62].

Of note, cytosolic PINK1 cannot promote mitophagy [63], suggesting that PINK1 pro-survival activity and its mitophagy-inducing role cannot be detached [50].

In a neuronal cell culture model for synucleinopathy, the downregulation of PINK1 enhanced alpha-synuclein aggregation and apoptosis.

## 4. Clinical Features and Genotype-Phenotype Correlation

As the other autosomal recessive EOPD forms, PINK1-associated PD is characterized by early onset of unilateral tremor, bradykinesia and rigidity that are often indistinguishable from other PD forms, especially PRKN and idiopathic PD. However, PINK1 patients often show uncommon characteristics which can help differential diagnosis, including hyperreflexia, dystonia at onset, early L-dopa induced dyskinesias, and psychiatric and behavioral disturbances.

The mean age of onset of PD symptoms is 33 years. Lower limb dystonia may be a presenting sign, or may develop during disease progression [18], and should raise the suspicion of PINK1-related PD. Nonmotor symptoms and sleep impairment are common in individuals with PINK1 type of young-onset PD [64]. Also, postural instability, hyperreflexia, cognitive-behavioral disturbances and especially psychiatric symptoms -including depression (17%), anxiety (10%), and psychosis—have been reported (see www.mdsgene.org, accessed on 10 July 2021).

Except for GBA-PINK1 is the PD-associated gene showing the highest rate of cognitive impairment, mainly concerning attention and executive functions [65], while the prevalence of depression is comparable between PINK1 patients and idiopathic PD.

Disease progression is usually benign and L-dopa response is often marked and sustained, with a high risk of developing L-dopa induced fluctuations throughout the disease [66].

Of note, although PINK1-related PD is associated with homozygous or compound heterozygous mutations, different heterozygous mutations have been reported in PD patients and healthy controls [18,19,20], but the effect of these mutations and their potential role as a risk factor for PD is far from been understood. In the past decade, some authors reported a higher prevalence of heterozygous PINK1 rare variants in PD patients when compared to healthy controls [18,21,67], thus suggesting that also heterozygous PINK1 mutations may predispose to PD. Particularly, Puschmann et al. [68] reported a genetic association between heterozygous PINK1 p.G411S mutations and PD, providing both structural and functional evidence for a negative effect of the mutant protein thus interfering with wild-type PINK1 kinase activity.

However, a recent wide assessment of PINK1 variants in 13,708 PD patients and 362,850 controls published by Krohn et al. [69] found no supporting evidence regarding the role of heterozygous PINK1 mutations as a risk factor for PD.

In conclusion, the spectrum of PINK1-associated phenotypes needs further investigation, especially regarding genotype-phenotype correlates; in fact, no correlation between the type of variant and age at onset, clinical presentation, or disease progression has yet been observed [70].

## 5. Current Therapeutic Approaches

Levodopa, the cardinal pharmacological treatment of PD, is effective in PINK1-associated PD. The classical parkinsonian symptoms (rigidity, bradykinesia and tremor) often show a dramatic and sustained response to levodopa and patients can be treated with relatively small doses for many years. PINK1 patients also benefit from dopamine agonists (ropinirole, pramipexole and rotigotine) which are frequently administered in the early stages of the disease, before levodopa is started [20,67,71,72,73,74,75,76,77,78]. As in classical PD, prolonged levodopa therapy invariably induces the appearance of motor fluctuations and dyskinesias. In some PINK1 patients, dyskinesias are reported early after levodopa is started, while others show a good response for many years before fluctuations and dyskinesias start. This difference does not seem to depend on disease duration and medication doses [18,20,64,67,71,72,73,74,75,78,79].

Patients with prominent and invalidating dyskinesias may benefit from amantadine [71,72,73].

Another side effect of antiparkinsonian medications, particularly dopamine-agonists, is impulse control disorder, a psychiatric condition characterized by a failure to resist an urge or a temptation, resulting in compulsive behaviors which can become self-harming. This complication has been reported in PINK1 patients also. It responds to dopamine-agonist withdrawal but in the most severe cases, antipsychotic drugs are needed [64,73].

One of the peculiarities of PINK1 related parkinsonism is the incidence of dystonia, which often presents in the early stages of the disease and more frequently involves the lower limbs. Some authors report a very good and sustained response of dystonia to small doses of levodopa, in some cases suggesting a misdiagnosis of dopa-responsive dystonia [71,79]. Other patients may not show the same responsiveness to levodopa and can be treated with anticholinergics (trihexyphenidyl hydrochloride and biperidene) but there are no reports of their efficacy [73,75,79].

The literature lacks information regarding the treatment of non-motor symptoms in PINK-1 patients, as well as on the pharmacological treatment of the advanced disease stages (i.e., with parenteral or intrajejunal drug administration).

The efficacy of surgical treatment by the implant of deep brain stimulation (DBS) electrodes, a well-established treatment for PD, has been explored only by few authors in PINK1 patients. The main anatomical targets in PD are the subthalamic nucleus (STN) and globus pallidus internus (GPi), with comparable results in terms of quality of life improvement [80]. Moro et al. [81] compare the short- and long-term surgical outcomes of bilateral STN-DBS in mutation-positive and -negative patients. The mutation-positive population included 11 Parkin and 1 PINK1 mutated patients. At baseline, the two groups were comparable except for a more severe postural instability and gait dysfunction in the mutated group. Overall, in mutation carriers the clinical response, measured as % change of unified Parkinson’s disease rating scale (UPDRS) motor score off-medication, was not superior to non–mutation carriers and authors suggest it could be limited by more advanced axial motor symptoms at a relatively early disease stage. The only patient with homozygous PINK1 mutation was a 61-year-old woman with long disease duration (30 years), showing a good response to DBS at short and long follow-up, but no further clinical details are given. Another report by Borellini et al. [82,83] describes a 49-year-old PINK1 female patient with a 19-year history of PD who underwent bilateral GPi-DBS. Short-term follow-up (2 months after activation), showed an improvement of quality of life mainly due to an improvement of lower limb dystonia and a complete resolution of motor fluctuations and particularly dyskinesias. At a longer follow-up, however, this improvement was partly reduced as lower limb dystonia re-emerged, poorly controlled either by pharmacological or electrical stimulation adjustments. Other few reports include heterozygous mutation carriers [84,85] and are not the object of this review.

Unlike pharmacological treatment, where the efficacy of common antiparkinsonian drugs has been established, the efficacy and long-term outcome of surgery still has to be elucidated. Further studies or case reports are needed to address this issue and which target gives the best results.

## 6. In Vitro Models

The first and major evidence about the complex role of mitophagy in cell metabolism mostly came from cultured cell lines treated with cytotoxic mitochondrial uncoupling or damaging agents.

Most of the data concerning in vivo models, instead, have been collected in the last few years, since the lack of strong methods to assess in vivo mitophagy has hampered progress in determining the exact role of mitophagy in normal physiology and various pathologic scenarios.

In 2010, Matsuda et al. [86] first described that Parkin translocation to OMM in damaged mitochondria was suppressed in PINK1-deficient murine embryonic fibroblasts (MEFs). Moreover, it was demonstrated that the elimination of dysfunctional mitochondria occurs through autophagy: in fact, autophagy-defective MEFs did not undergo PINK1/Parkin-mediated degradation.

In cell culture, PINK1 can be activated through mitochondrial depolarization induced by mitochondrial uncoupling agents such as antimycin A/oligomycin (A/O). This results in the recruitment and activation of Parkin at the OMM [22]. PINK1 then phosphorylates Parkin at its Ser65 residue as well as the equivalent residue of ubiquitin, which are both required for complete activation of Parkin E3 ligase activity [40,41]. Upon activation, Parkin ubiquitylates multiple substrates at the OMM, leading to both de novo assembly and elongation of existing ubiquitin chains that are, in turn, phosphorylated by PINK1, generating a feed-forward enhancement of Parkin activation and mitochondrial ubiquitylation that ultimately leads to mitophagy [36,87]. 

Recent advances in human induced pluripotent stem cells (iPSCs) and human embryonic stem cells (ESCs) research constitute a new opportunity for disease modeling [88], giving the possibility to generate PD models directly in patient-specific and disease-relevant human cells, such as midbrain dopaminergic neurons, which play a key role in PD-associated neurodegeneration.

Particularly, iPSCs differentiation has enabled the generation of iPSC-derived neurons from PARK2 (Parkin) patients, which exhibited aberrant mitochondrial morphology and altered homeostasis, associated with increased oxidative stress and aberrant alpha-synuclein accumulation [89].

Thanks to the advances in cell reprogramming, in 2016 Young Chung et al. [90] first demonstrated that the identification of disease-related phenotypes in PD-patient-specific iPSCs-derived midbrain dopaminergic neurons depends on the type of differentiation protocol utilized. Of note, they found that both PINK1 and Parkin patient-derived midbrain dopaminergic neurons displayed higher levels of alpha-synuclein expression at the gene and protein levels, aberrant mitochondrial morphology, and increased vulnerability to mitochondrial venoms. Moreover, midbrain DA neurons exhibited different disease-related phenotypes depending on the type of differentiation protocol utilized, thus stressing the critical role of iPSC-derived midbrain neurons in determining disease-relevant phenotypes for in vitro models. Also, this finding suggests the existence of a pathogenetic loop involving mitochondrial dysfunction, increased susceptibility, and abnormal neurotransmitter homeostasis.

Another crucial theme regarding PINK1-PRKN mutated PD patients is the associated iron accumulation ad a result of dysfunctional mitophagy. In 2020, Key J. et al. [91] demonstrated that ferritin superfamily and nucleotide surveillance regulation are strictly modulated by PINK1 in mouse and patient cells, exploring the possibility to employ iron chelator drugs in this subgroup of PD patients.

Yamaguchi et al. [92] recently purposed a semi-automatic high-throughput assay system for quantitative detection of disease-specific phenotypes in iPSCs-derived DA neurons of patients with familial PD having Parkin or PINK1 mutations which exhibit abnormal mitochondrial homeostasis, thus in order to screen potential therapeutic drugs which may restore impaired mitochondrial homeostasis as observed in PINK1/Parkin neurons. They identified 4 candidate drugs (MRS 1220, Tranylcypromine, Flunarizine and Bromocriptine) that partially rescued the phenotypes in DA neurons and then tested their efficacy using both in vivo and in vitro models, confirming their ability to partially rescue phenotypes in Drosophila PD models and iPSCs derived from patients with idiopathic PD, especially in terms of mitophagy and ATP production. Thus, the proposed system has a great potential for identifying potential effective drugs for both familial and idiopathic PD.

## 7. In Vivo Models

As mentioned above, despite the wealth of mechanistic information on PINK1/Parkin role in mitophagy in cultured cells, to date evidence for their relevance in vivo is scarce and still represents a major challenge [93,94]. Ubiquitin phosphorylated at Ser65-which can be considered as a biomarker of PINK1 activity-accumulates in brains from elderly human subjects [95], and mice with defective mitochondrial DNA proofreading [96], suggesting that PINK1-mediated mitophagy is induced by both aging and accumulation of mitochondrial damage.

The fruit fly Drosophila melanogaster was the first in vivo model used to demonstrate Parkin role in mitochondrial homeostasis [97], and to detect the existence of a pathway involving both PINK1 and Parkin, which aims to promote the selective autophagic degradation of damaged mitochondria [98,99,100].

Clark et al. [99] first described that the *Drosophila* PINK1 homologue deficiency induces male sterility due to deficit in mitochondrial functioning during spermatogenesis, apoptotic muscle degeneration with poor flight performance, abnormal mitochondrial cristae morphology, and increased vulnerability to multiple stresses including oxidative stress after exposure to both paraquat, a free radical inducer, and rotenone, a molecule which counteracts complex I activity.

Notably, transgenic expression of human PINK1 in the *Drosophila* revealed to be able to rescue male fertility and normal mitochondrial morphology in some of the PINK1 mutants, suggesting a certain grade of functional homology between human and *Drosophila* PINK1. Mitochondrial morphology and muscle and dopaminergic neuron degeneration in PINK1 mutants can also be rescued by Parkin overexpression [101], whereas overexpression of PINK1 does not similarly recover Parkin-mutant phenotypes.

Moreover, Parkin and PINK1 mutants are very similar in their mitochondrial phenotypes [97,98], and Parkin overexpression in PINK1 mutants has similar effects to the expression of human PINK1 in terms of restoring male fertility, flight muscle integrity and normal mitochondrial function, thus pointing toward the existence of a PINK1/Parkin pathway, with PINK1 acting-at least in part—upstream of Parkin [99]. In 2008, Todd and Staveley [102] demonstrated that the overexpression of PINK1 in *Drosophila* can protect against alpha-syn induced phenotype.

Another important study on *Drosophila* [103] provided a genetic model of mitochondrial protein misfolding, proving that PINK1 and Parkin mutations display higher levels of misfolded components of mitochondrial respiratory complexes, whose clearance is usually promoted by Parkin-mediated enhancement of autophagy processes. The accumulation of misfolded proteins, in turn, leads to global mitochondrial dysfunction and subsequent fragmentation.

To further support this hypothesis, Vincow et al., 2013 [104] proposed an innovative proteomic-based assay to monitor the turnover of mitochondrial proteins by using quantitative mass spectrometry in *Drosophila* models, providing the first evidence of PINK1 and Parkin role in physiological mitochondrial turnover: in fact, they found out that the half-life of many mitochondrial proteins was significantly prolonged in Parkin null mutants than in wild-type flies, and was similar—although less severe than—to the one observed in the case of general autophagy-defective flies (Atg7 null mutants). This relationship was specific to mitochondrial proteins: no correlation occurred between the effects of Parkin and Atg7 mutations on the turnover of proteins from other organellar targets of autophagy. This evidence suggests that the absence of Parkin affects autophagy-related mitochondrial proteins turnover, generating an abnormal accumulation of misfolded and potentially dysfunctional proteins in vivo. However, results from PINK1 mutants were more complex, suggesting a mitophagy deficit largely compensated by the activation of an alternative turnover pathway.

Also, in the same study Vinchow et al. found out that both Parkin and PINK1 are implicated in a process of selective non-mitophagic turnover of mitochondrial respiratory chain subunits such as Complex I, which may explain the respiratory chain impairment observed in both familial and sporadic PD patients.

Given the importance of Complex I in mitochondrial bioenergetics, different studies demonstrated the presence of alterations in the redox state of the Complex I substrate nicotinamide adenine dinucleotide (NAD+) in Drosophila pink1 mutants, with consequent impairment in mitochondrial energy production [105,106]. Lehmann et al. (2017) [107] showed that both a diet supplemented with the NAD+ precursor nicotinamide and the inhibition of NAD+-dependent enzymes—such as poly(ADP-ribose) polymerases (PARPs), which are major NAD+-consuming enzymes involved in nuclear DNA repair and compete with mitochondria for NAD+—rescued mitochondrial defects and protected neurons from degeneration.

Also, mitochondrial bioenergetics and metabolism are widely known to be strictly dependent from calcium signaling, with calcium being involved in many mitochondrial functions including oxidative phosphorylation and bioenergetic maintenance. In this setting, DA neurons are likely to be more susceptible than other cell types to calcium homeostasis disruption, given their intrinsic pacemaking activity [108]. One of the main mechanisms through which calcium enters mitochondria is the ER-mitochondria transport, which takes place on the ER-mitochondria contact sites (ERMCSs) where calcium levels are high [109]. Miro protein, a well-known protein involved in mitochondrial axonal transport, has also shown to play a pivotal role in regulating mitochondrial Ca influx in both a transport-dependent and -independent manner [110,111]. Nevertheless, PINK1 has showed to negatively regulate Miro level in DA neurons. After previous studies already proving that Miro protein levels are increased in PINK1 mutants [112,113], Lee et al. [108] found that PINK1 mutant DA neurons of PD model flies show increased ERMCSs as a result of an increasing in Miro protein level, thus increasing mitochondrial calcium uptake and, ultimately, mitochondrial enlargement and neuronal death. In this context, it becomes clear that mitochondrial calcium homeostasis and PINK1/Miro/ERMCSs signaling modulation may offer a potential therapeutic avenue in PD.

Of note, among the great number of co-substrates of PINK1 and Parkin involved in mitochondrial quality control, the pathogenic substrate PARIS (ZNF46) is known to be strictly regulated by PINK1-mediated phosphorylation and Parkin-dependent ubiquitination [114,115]. Pirooznia et al. [116] created Drosophila models of PARIS accumulation showing that expression of PARIS leads to selective and progressive age-dependent loss of DA neurons, with consequent alteration in locomotion and climbing performance; these deficits are rescued by overexpression of Parkin or PINK1, while silencing of PINK1 or Parkin leads to a DA phenotype resembling the one of PARIS transgenic flies and it moreover worsens the PARIS phenotype. The authors showed that the mechanism underlying PARIS neurotoxicity is mediated by an alteration in mitochondrial biogenesis, thus supporting once more the role of altered mitochondrial biogenesis in DA neurotoxicity under conditions of PINK1/Parkin deficiency.

Another recently studied pathway in PINK1/Parkin models is the cyclic GMP–AMP synthase (cGAS)–stimulator of interferon genes (STING) pathway, a system implicated in a wide range of cellular processes including autophagy [117], and thus being investigated as a potential player in neurodegenerative diseases such as PD. Particularly, Sliter et al. [118] found out that co-deletion of STING was able to rescue the motor deficit and neuronal loss observed in in Parkin-deficient mice. However, a more recent study by Lee et al. [119] Drosophila models showed that the only loss of STING was not sufficient to rescue the behavioral or mitochondrial phenotypes in Drosophila Pink1/Parkin mutants.

Some recent studies also focused on modeling non-motor symptoms of PD in flies carrying loss-of-function mutations in PINK1 and Parkin, revealing that both mutant genotypes displayed reduced learning and memory performances, as well as a weakening of circadian rhythms, suggesting novel mechanisms of action of these disease-causing genes and providing support for the idea that cognitive and circadian dysregulation is an intrinsic aspect of PD rather than a side effect of medication or linked to environmental factors [120].

In 2018, a study published by Cornelissen et al. [121] found that mitophagy occurs in *Drosophila* flight muscle and dopaminergic neurons in vivo, even in the absence of exogenous mitochondrial toxins. Mitophagy in these cells increases with aging, but this rise is abolished in PINK1 or Parkin mutants, thus suggesting a possible explanation for the age-dependent neurodegeneration that occurs in patients who carry a loss-of-function mutation in at least one of these two genes.

Han et al. recently identified Drp1 as a new substrate of PINK1 and a novel, Parkin-independent way through which PINK1 regulates mitochondrial fission and autophagy, thus further strengthening the linking between PINK1-mediated Drp1S616 phosphorylation and PD pathogenesis [122]. Moreover, studies on the *Drosophila* phosphatidylinositol 4-kinase IIIβ homologue Four wheel drive (fwd), a pro-fission factor which regulates mitochondrial fission in vivo, showed that this kinase acts downstream of Drp1, and that fwd mutants appears to have similarities with Pink1/Parkin mutants, while fwd overexpression can rescue mitochondrial disfunction in Pink1/Parkin mutants [123].

Another emerging role of PINK1 concerns tissue growth and tumorigenesis; it is known that PINK1 is downregulated in PTEN-mutated tumor cells. A recent study by Han et al. [124] reported that PINK1 deficiency causes Parkin-independent growth impairment, and that PTEN activity reduction induces a sort of “wasting-like syndrome” with impaired systemic growth through a reduction in pink1 expression. Moreover, expression of PINK1 fully rescues growth defects in Drosophila models.

Nevertheless, studies in Drosophila still show some limitations and unsolved issues [94], such as: (a) PINK1 and Parkin mutants show flight muscle disruption before mitochondrial damage, which may suggest that PINK1 and Parkin role in flight muscle may not be related to mitophagy; (b) In addition, the evidence that Parkin overexpression can rescue mitochondrial dysfunction in PINK1-deficient goes against the irreplaceable role of PINK1 in mitophagy as showed in HeLa cells [125].

The other relevant in vivo PINK1 animal model is the mice. Wang et al. [126,127] first showed that PINK1 mice mutants are defective in mitochondrial membrane potential maintenance, and exhibit higher levels of ROS formation in SN dopaminergic neurons. In mice, the germline deletion of the PINK1 gene significantly impaired mitochondrial function causing hypersensitivity to oxidative stress, with a certain selectivity for dopaminergic circuitry [128]. The recently described mito-QC and mt-Keima mouse models generated by respectively McWilliams et al. [129] and Sun et al. [130] have first unraveled the striking and widely heterogeneous nature of basal mitophagy.

The first great limitation of mice models is that PINK1/Parkin knock-out mice do not display any significant neurological or behavioral disruption, which downsizes the importance of this pathway in cell survival. On the other hand, an important piece of evidence is that “post-natal conditional Parkin k.o.” mice results in loss of nigral DA neurons, suggesting a possible compensatory mechanism to be responsible for neuronal integrity preservation in germline k.o. [131]. It is still not clear how this mechanism could work, but Parkin-independent mitophagy pathways may play a role in compensating Parkin deficiency; anyway, this evidence invariably underlines an inconsistency between mice and humans, since PINK1/Parkin loss leads to marked, early-onset neurodegeneration in human.

Another important evidence regarding the role of PINK1 and Parkin in mitochondrial homeostasis is provided by the finding that PINK1/Parkin germline k.o. mice do not display any sign of neurodegeneration, whereas the presence of an add-on second hit due to the expression of a proof-reading defective mtDNA polymerase (POLG)—which cause accumulation of altered mitochondria due to a higher mtDNA mutational rate (the so-called “mutator mice” model)—induces selective age-dependent nigral dopaminergic neuron degeneration and levodopa-responsive motor dysfunction under conditions of constitutive mitochondrial stress [96,132], thus proving a certain role of Parkin in mitochondrial quality control in vivo; nevertheless, surprisingly, loss of Parkin induces mitochondrial dysfunction but seems not to affect overall levels of mtDNA somatic mutations, as if the absence of Parkin did not have to deal with the lack of mutated mitochondria degradation.

Also, AMP kinase (AMPK), a master regulator of cellular energy homeostasis, has been studied in mice models in order to better clarify its distribution and role in the mammalian brain: Hang et al. [133] recently found that phosphorylated AMPK is highly expressed in the ventral midbrain of non-mutated mice, where SN dopaminergic neurons reside, and relatively underexpressed in other cerebral areas; of note, this physiological midbrain phospho-AMPK overexpression is selectively disrupted in Parkin-deficient as well as in PINK1-deficient mice.

In 2015, Gispert et al. [134] crossed mice with A53T-SNCA overexpression and with PINK1 deletion; they found that double mutant mice had a reduced lifespan and confirmed that SNCA-triggered neurotoxicity is exacerbated by the absence of PINK1.

Despite all the efforts in the last years, the physiological significance of Parkin Ser65 phosphorylation by PINK1 in vivo in mammals remains poorly clear. To test the importance of Parkin Ser65 phosphorylation in vivo, McWilliams et al. (2018) [135] recently generated a Parkin Ser65Ala (S65A) knock-in mouse model in which the codon encoding Parkin Ser65 was altered to prevent its phosphorylation by PINK1. They observed endogenous Parkin Ser65 phosphorylation and activation in mature primary neurons following mitochondrial depolarization and revealed this is disrupted in Parkin S65A/S65A neurons. Regarding the clinical phenotype, Parkin S65A/S65A mice showed selective motor disruption but no clear neurodegeneration or nigrostriatal mitophagy impairment. Moreover, Parkin S65A/S65A mutants cannot be activated by PINK1, thus pointing toward an actual critical role of Parkin Ser65 phosphorylation in mitochondrial homeostasis. However, although undeniable, the precise role of PINK1-Parkin signaling in mitophagy in vivo remains unclear.

In 2018, Zhi et al. [136] measured DA release in acute striatal slices from both PINK1 KO and WT mice at different ages, and found that dorsal striatum of PINK1 KO mice had significantly lower basal mitochondria respiration compared with that of controls, and also showed less DA release, thus suggesting that the impaired DA release may be mostly related to mitochondrial impairment and lower ATP levels.

## 8. Future Therapeutic Targets

Several gene-targeted therapies for PD are currently in development [137], particularly for PD patients carrying glucosylceramidase β (GBA) or leucine-rich repeat kinase-2 (LRRK2) pathogenic variants (www.clinicaltrials.gov, accessed on 10 July 2021).

Given the critical relevance of mitophagy and mitochondrial dysfunction in PD and particularly in PINK1-related PD, restoring normal mitophagy by targeting the PINK1/Parkin pathway is an emerging promising therapeutic approach for the disease. Several small molecule drugs to enhance mitophagy and biomarkers for mitochondrial dysfunction are in preclinical development [138,139]. However, we still do not dispose of pharmacological agents that selectively modulate mitophagy [140,141].

Several potential mitophagy enhancers have been evaluated so far.

In 2014, East et al. [142] found that both trifluorocarbonylcyanide phenylhydrazone and the combination of antimycin/oligomycin could be used to trigger mitophagy; nevertheless, their effects were toxic and non-specific.

The mitochondrial receptor Nip3-like protein X (Nix) can be seen as an alternative mediator of mitophagy, thus representing another potential promising target for PINK1/Parkin-related PD treatment when PINK1-Parkin mediated mitophagy is impaired; Koentjoro et al. [143] found that Nix overexpression rescues mitochondrial function in fibroblast lines derived from homozygous mutated PINK1 patients.

Moreover, mitochondrial damaging activates several events including translocation of specific molecules called “eat-me” signals which accumulate on the mitochondrial outer membrane and undergo Parkin-mediated ubiquitination, thus enabling the lysosomal-mediated mitochondrial degradation, which occurs during the mitophagic process. Among these molecules, the matrix proteins NIPSNAP1 (nipsnap homolog 1) and NIPSNAP2 (nipsnap homolog 2) have been shown to contribute to PINK1-PRKN-dependent mitophagy [144], and their PRKN-dependent ubiquitination may be an early and crucial step in the recruitment of autophagy receptors. Conversely, as *Abudu* et al. found [145], Zebrafish lacking Nipsap1 have reduced brain mitophagy, increased reactive oxygen species (ROS) levels and loss of DA neurons, thus confirming results already obtained from cellular models and suggesting a potential role of Nipsnap1 in rescuing PINK1-PRKN-dependent mitophagy in PINK1 phenotypes.

**Figure 2:**
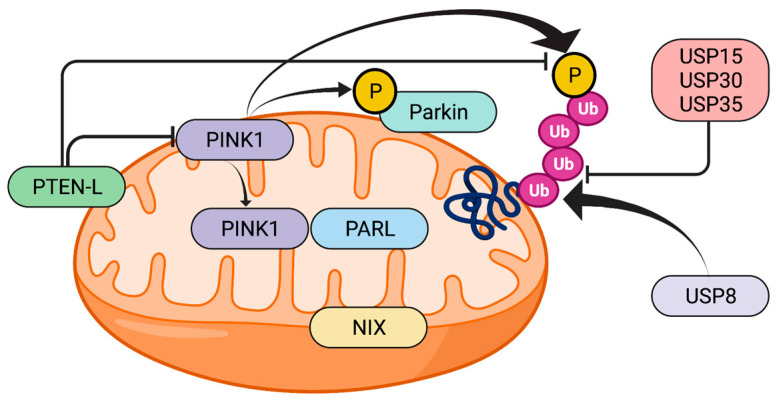
Schematic representation of PINK1-Parkin mitochondrial axis and some of the above-mentioned main potential therapeutic targets. PTEN-L, phosphate and tension homology deleted on chromosome ten-long; Ub, ubiquitin; USP, ubiquitin specific protease; NIX, Nip3-like protein X; PARL, presenilins-associated rhomboid-like protein. Created with BioRender.com.

Another potential way to activate mitophagy is to modulate the PINK1-Parkin-Ub feedforward loop in many different ways. Of note, PTEN-L inhibitors can prevent PTEN-mediated dephosphorylation of Parkin and ubiquitin, thus allowing mitophagy activation [44]. PTEN-L role in mitophagy represents a key point for future in vivo studies concerning PINK1 and Parkin PD targeted treatment [39,44]. Moreover, the DUB USP30, USP35, and USP15 were found to counteract Parkin activity, so their inhibition can be used to promote mitophagy [146,147,148]. On the contrary, USP8 regulates mitophagy positively by removing K6-linked ubiquitin conjugates from Parkin [149] (Figure 2).

Likewise, since the mitochondrial dysfunction is strictly related to the accumulation of ROS and rising of oxidative stress, applications of antioxidants could also be a promising avenue in treating PD; however, although many antioxidants, such as creatine, vitamin E, coenzyme Q10, pioglitazone, melatonin, and desferrioxamine, have been tested in clinical trials, none of them has demonstrated conclusive evidence to revert or improve neurodegeneration in PD patients. Difficulties in clinical trials may be due to the long-standing progression of neurodegeneration, lack of biomarkers for the premotor stage of PD, and inadequate drug delivery across the blood-brain barrier, so further studies on PD patients are needed to overcome these limitations.

Another important consideration is that mitochondrial dysfunction is known to be impaired in PD patients beyond those with genetic mutations in the PINK1/Parkin pathway, so mitophagy enhancers may have a broader spectrum of application. However, the removal of too many mitochondria may also have a paradoxical negative effect, since the complex pathway of mitophagy is intricately regulated and connected to many other networks, such as lysosomal function and inflammation. Hence, it becomes necessary to stratify PD patients who exhibit mitochondrial dysfunction that may most benefit from this kind of treatment and identify biomarkers for mitochondrial dysfunctions to carefully monitor therapeutic responses [149]. Moreover, another critical point is to preferentially target downstream autophagy-lysosomal pathway components rather than use macroautophagy-enhancing agents, since their lack of selectivity may cause several dose-dependent side-effects [141].

## 9. Conclusions

In this review, we extensively illustrate the intricate machinery of mitophagy and the fundamental role of the PINK1-Parkin axis in the pathogenesis of PD, providing an overview of the experimental evidence gained from the realization of both in vitro and in vivo models.

Assuming that PINK1 and Parkin are the master regulators of basal mitophagy, it is difficult to reconcile the prevalence of basal mitophagy in several tissues with the selective involvement of mitochondria of nigral dopaminergic neurons, as observed in PD patients.

Further studies will be needed to better clarify the precise contribution of PINK1-Parkin axis to basal mitophagy in vivo to develop therapeutic agents able to slow the pathological and clinical progression of the disease.

## References

[1] Pissadaki E.K., Bolam J.P. (2013). The energy cost of action potential propagation in dopamine neurons: Clues to susceptibility in Parkinson’s disease. Front. Comput. Neurosci..

[2] Guzman R.E., Schwarz Y.N., Rettig J., Bruns D. (2010). SNARE Force Synchronizes Synaptic Vesicle Fusion and Controls the Kinetics of Quantal Synaptic Transmission. J. Neurosci..

[3] Moore D.J., West A.B., Dawson V.L., Dawson T.M. (2005). Molecular pathophysiology Of Parkinson’s disease. Annu. Rev. Neurosci..

[4] Narendra D., Tanaka A., Suen D.-F., Youle R.J. (2008). Parkin is recruited selectively to impaired mitochondria and promotes their autophagy. J. Cell Biol..

[5] Pickrell A.M., Youle R.J. (2015). The Roles of PINK1, Parkin, and Mitochondrial Fidelity in Parkinson’s Disease. Neuron.

[6] Yamano K., Matsuda N., Tanaka K. (2016). The ubiquitin signal and autophagy: An orchestrated dance leading to mitochondrial degradation. EMBO Rep..

[7] Klein C., Schlossmacher M.G. (2007). Parkinson disease, 10 years after its genetic revolution: Multiple clues to a complex disorder. Neurology.

[8] Zhang B.-R., Hu Z.-X., Yin X.-Z., Cai M., Zhao G.-H., Liu Z.-R., Luo W. (2010). Mutation analysis of parkin and PINK1 genes in early-onset Parkinson’s disease in China. Neurosci. Lett..

[9] Unoki M., Nakamura Y. (2001). Growth-suppressive effects of BPOZ and EGR2, two genes involved in the PTEN signaling pathway. Oncogene.

[10] Valente E.M., Bentivoglio A.R., Dixon P.H., Ferraris A., Ialongo T., Frontali M., Albanese A., Wood N.W. (2001). Localization of a Novel Locus for Autosomal Recessive Early-Onset Parkinsonism, PARK6, on Human Chromosome 1p35-p36. Am. J. Hum. Genet..

[11] Valente E.M., Brancati F., Ferraris A., Graham E.A., Davis M.B., Breteler M.M.B., Gasser T., Bonifati V., Bentivoglio A.R., De Michele G. (2002). PARK6-Linked Parkinsonism Occurs in Several European Families. Ann. Neurol..

[12] Valente E.M., Abou-Sleiman P.M., Caputo V., Muqit M.M.K., Harvey K., Gispert S., Ali Z., Del Turco D., Bentivoglio A.R., Healy D.G. (2004). Hereditary Early-Onset Parkinson’s Disease Caused by Mutations in PINK1. Science.

[13] Kawajiri S., Saiki S., Sato S., Hattori N. (2011). Genetic mutations and functions of PINK1. Trends Pharmacol. Sci..

[14] Kasten M., Hartmann C., Hampf J., Schaake S., Westenberger A., Vollstedt E.-J., Balck A., Domingo A., Vulinovic F., Dulovic M. (2018). Genotype-Phenotype Relations for the Parkinson’s Disease Genes Parkin, PINK1, DJ1: MDSGene Systematic Review. Mov. Disord..

[15] Djarmati A., Hedrich K., Svetel M., Lohnau T., Schwinger E., Romac S., Pramstaller P.P., Kostić V., Klein C. (2006). HeterozygousPINK1 mutations: A susceptibility factor for Parkinson disease?. Mov. Disord..

[16] Siuda J., Jasinska-Myga B., Boczarska-Jedynak M., Opala G., Fiesel F.C., Moussaud-Lamodière E.L., Scarffe L.A., Dawson V.L., Ross O.A., Springer W. (2014). Early-onset Parkinson’s disease due to PINK1 p.Q456X mutation—Clinical and functional study. Parkinsonism Relat. Disord..

[17] Grünewald A., Breedveld G.J., Lohmann-Hedrich K., Rohé C.F., König I.R., Hagenah J., Vanacore N., Meco G., Antonini A., Goldwurm S. (2007). Biological effects of the PINK1 c.1366C>T mutation: Implications in Parkinson disease pathogenesis. Neurogenetics.

[18] Bonifati V., Rohe C.F., Breedveld G.J., Fabrizio E., De Mari M., Tassorelli C., Tavella A., Marconi R., Nicholl D.J., Chien H.F. (2005). Early-onset parkinsonism associated with PINK1 mutations: Frequency, genotypes, and phenotypes. Neurology.

[19] Choi J.M., Woo M.S., Ma H.-I., Kang S.Y., Sung Y.-H., Yong S.W., Chung S.J., Kim J.-S., Shin H.-W., Lyoo C.H. (2008). Analysis of PARK genes in a Korean cohort of early-onset Parkinson disease. Neurogenetics.

[20] Weng Y.-H., Chou Y.-H.W., Wu W.-S., Lin K.-J., Chang H.-C., Yen T.-C., Chen R.-S., Wey S.-P., Lu C.-S. (2007). PINK1 mutation in Taiwanese early-onset parkinsonism. J. Neurol..

[21] Brooks J., Ding J., Sánchez J.S., Paisan-Ruiz C., Singleton A.B., Scholz S. (2009). Parkin and PINK1 mutations in early-onset Parkinson’s disease: Comprehensive screening in publicly available cases and control. J. Med. Genet..

[22] McWilliams T.G., Muqit M.M. (2017). PINK1 and Parkin: Emerging themes in mitochondrial homeostasis. Curr. Opin. Cell Biol..

[23] Winklhofer K.F. (2014). Parkin and mitochondrial quality control: Toward assembling the puzzle. Trends Cell Biol..

[24] Durcan T.M., Fon E.A. (2015). USP8 and PARK2/parkin-mediated mitophagy. Autophagy.

[25] Eiyama A., Okamoto K. (2015). PINK1/Parkin-mediated mitophagy in mammalian cells. Curr. Opin. Cell Biol..

[26] Daou S., Sicheri F. (2017). Vivid views of the PINK1 protein. Nat. Cell Biol..

[27] Kumar A., Tamjar J., Waddell A.D., Woodroof H.I., Raimi O.G., Shaw A.M., Peggie M., Muqit M.M., Van Aalten D.M. (2017). Structure of PINK1 and mechanisms of Parkinson’s disease-associated mutations. eLife.

[28] Schubert A.F., Gladkova C., Pardon E., Wagstaff J.L., Freund S.M.V., Steyaert J., Maslen S.L., Komander D. (2017). Structure of PINK1 in complex with its substrate ubiquitin. Nat. Cell Biol..

[29] Bender A., Krishnan K.J., Morris C.M., Taylor G.A., Reeve A.K., Perry R.H., Jaros E., Hersheson J.S., Betts J., Klopstock T. (2006). High levels of mitochondrial DNA deletions in substantia nigra neurons in aging and Parkinson disease. Nat. Genet..

[30] Kraytsberg Y., Kudryavtseva E., McKee A.C., Geula C., Kowall N.W., Khrapko K. (2006). Mitochondrial DNA deletions are abundant and cause functional impairment in aged human substantia nigra neurons. Nat. Genet..

[31] Langston J.W., Ballard P.A. (1983). Parkinson’s Disease in a Chemist Working with 1-Methyl-4-Phenyl-L,2,5,6-Tetrahydropyridine. N. Engl. J. Med..

[32] Schapira A., Cooper J., Dexter D., Jenner P., Clark J., Marsden C. (1989). Mitochondrial complex I deficiency in Parkinson’s disease. Lancet.

[33] Greene A.W., Grenier K., Aguileta M.A., Muise S., Farazifard R., Haque M.E., McBride H.M., Park D.S., Fon E.A. (2012). Mitochondrial processing peptidase regulates PINK1 processing, import and Parkin recruitment. EMBO Rep..

[34] Deas E., Plun-Favreau H., Gandhi S., Desmond H., Kjaer S., Loh S.H., Renton A.E., Harvey R., Whitworth A., Martins L.M. (2010). PINK1 cleavage at position A103 by the mitochondrial protease PARL. Hum. Mol. Genet..

[35] Yamano K., Youle R.J. (2013). PINK1 is degraded through the N-end rule pathway. Autophagy.

[36] Lazarou M., Jin S.M., Kane L.A., Youle R.J. (2012). Role of PINK1 Binding to the TOM Complex and Alternate Intracellular Membranes in Recruitment and Activation of the E3 Ligase Parkin. Dev. Cell.

[37] Kondapalli C., Kazlauskaite A., Zhang N., Woodroof H.I., Campbell D.G., Gourlay R., Burchell L., Walden H., Macartney T.J., Deak M. (2012). PINK1 is activated by mitochondrial membrane potential depolarization and stimulates Parkin E3 ligase activity by phosphorylating Serine 65. Open Biol..

[38] Chen Y., Dorn G.W. (2013). PINK1-Phosphorylated Mitofusin 2 Is a Parkin Receptor for Culling Damaged Mitochondria. Science.

[39] Tanaka K. (2020). The PINK1–Parkin axis: An Overview. Neurosci. Res..

[40] Kane L.A., Lazarou M., Fogel A.I., Li Y., Yamano K., Sarraf S., Banerjee S., Youle R.J. (2014). PINK1 phosphorylates ubiquitin to activate Parkin E3 ubiquitin ligase activity. J. Cell Biol..

[41] Kazlauskaite A., Martinez-Torres R.J., Wilkie S., Kumar A., Peltier J., Gonzalez A., Johnson C., Zhang J., Hope A.G., Peggie M. (2015). Binding to serine 65-phosphorylated ubiquitin primes Parkin for optimal PINK 1-dependent phosphorylation and activation. EMBO Rep..

[42] Sauvé V., Lilov A., Seirafi M., Vranas M., Rasool S., Kozlov G., Sprules T., Wang J., Trempe J., Gehring K. (2015). A Ubl/ubiquitin switch in the activation of Parkin. EMBO J..

[43] Koyano F., Okatsu K., Kosako H., Tamura Y., Go E., Kimura M., Kimura Y., Tsuchiya H., Yoshihara H., Hirokawa T. (2014). Ubiquitin is phosphorylated by PINK1 to activate parkin. Nat. Cell Biol..

[44] Wang L., Cho Y.-L., Tang Y., Wang J., Park J.-E., Wu Y., Wang C., Tong Y., Chawla R., Zhang J. (2018). PTEN-L is a novel protein phosphatase for ubiquitin dephosphorylation to inhibit PINK1–Parkin-mediated mitophagy. Cell Res..

[45] Thomas R.E., Andrews L.A., Burman J.L., Lin W.-Y., Pallanck L.J. (2014). PINK1-Parkin Pathway Activity Is Regulated by Degradation of PINK1 in the Mitochondrial Matrix. PLoS Genet..

[46] Poole A.C., Thomas R.E., Yu S., Vincow E.S., Pallanck L. (2010). The Mitochondrial Fusion-Promoting Factor Mitofusin Is a Substrate of the PINK1/Parkin Pathway. PLoS ONE.

[47] Gegg M.E., Schapira A.H. (2011). PINK1-parkin-dependent mitophagy involves ubiquitination of mitofusins 1 and 2: Implications for Parkinson disease pathogenesis. Autophagy.

[48] Morais V.A., Verstreken P., Roethig A., Smet J., Snellinx A., Vanbrabant M., Haddad D., Frezza C., Mandemakers W., Vogt-Weisenhorn D. (2009). Parkinson’s disease mutations in PINK1 result in decreased Complex I activity and deficient synaptic function. EMBO Mol. Med..

[49] Morais V.A., Haddad D., Craessaerts K., De Bock P.-J., Swerts J., Vilain S., Aerts L., Overbergh L., Grünewald A., Seibler P. (2014). PINK1 Loss-of-Function Mutations Affect Mitochondrial Complex I Activity via NdufA10 Ubiquinone Uncoupling. Science.

[50] Voigt A., Berlemann L.A., Winklhofer K.F. (2016). The mitochondrial kinase PINK1: Functions beyond mitophagy. J. Neurochem..

[51] Soubannier V., McLelland G.-L., Zunino R., Braschi E., Rippstein P., Fon E.A., McBride H.M. (2012). A Vesicular Transport Pathway Shuttles Cargo from Mitochondria to Lysosomes. Curr. Biol..

[52] Braschi E., Goyon V., Zunino R., Mohanty A., Xu L., McBride H. (2010). Vps35 Mediates Vesicle Transport between the Mitochondria and Peroxisomes. Curr. Biol..

[53] Vilariño-Güell C., Wider C., Ross O., Dachsel J.C., Kachergus J.M., Lincoln S.J., Soto-Ortolaza A.I., Cobb S.A., Wilhoite G.J., Bacon J.A. (2011). VPS35 Mutations in Parkinson Disease. Am. J. Hum. Genet..

[54] Zimprich A., Benet-Pagès A., Struhal W., Graf E., Eck S.H., Offman M.N., Haubenberger D., Spielberger S., Schulte E.C., Lichtner P. (2011). A Mutation in VPS35, Encoding a Subunit of the Retromer Complex, Causes Late-Onset Parkinson Disease. Am. J. Hum. Genet..

[55] Charman M., Kennedy B.E., Osborne N., Karten B. (2010). MLN64 mediates egress of cholesterol from endosomes to mitochondria in the absence of functional Niemann-Pick Type C1 protein. J. Lipid Res..

[56] Elbaz-Alon Y., Rosenfeld-Gur E., Shinder V., Futerman A., Geiger T., Schuldiner M. (2014). A Dynamic Interface between Vacuoles and Mitochondria in Yeast. Dev. Cell.

[57] Wong Y.C., Ysselstein D., Krainc D. (2018). Mitochondria–lysosome contacts regulate mitochondrial fission via RAB7 GTP hydrolysis. Nature.

[58] Demers-Lamarche J., Guillebaud G., Tlili M., Todkar K., Bélanger N., Grondin M., Nguyen A.P., Michel J., Germain M. (2016). Loss of Mitochondrial Function Impairs Lysosomes. J. Biol. Chem..

[59] Todkar K., Chikhi L., Germain M. (2019). Mitochondrial interaction with the endosomal compartment in endocytosis and mitochondrial transfer. Mitochondrion.

[60] Petit A., Kawarai T., Paitel E., Sanjo N., Maj M., Scheid M., Chen F., Gu Y., Hasegawa H., Salehi-Rad S. (2005). Wild-Type PINK1 Prevents Basal and Induced Neuronal Apoptosis, a Protective Effect Abrogated by Parkinson Disease-Related Mutations. J. Biol. Chem..

[61] Klinkenberg M., Thurow N., Gispert S., Ricciardi F., Eich F., Prehn J., Auburger G., Kögel D. (2010). Enhanced vulnerability of PARK6 patient skin fibroblasts to apoptosis induced by proteasomal stress. Neuroscience.

[62] Haque M.E., Mount M.P., Safarpour F., Abdel-Messih E., Callaghan S., Mazerolle C., Kitada T., Slack R., Wallace V., Shen J. (2012). Inactivation of Pink1 Gene in Vivo Sensitizes Dopamine-Producing Neurons to 1-Methyl-4-phenyl-1,2,3,6-tetrahydropyridine (MPTP) and Can Be Rescued by Autosomal Recessive Parkinson Disease Genes, Parkin or DJ-1. J. Biol. Chem..

[63] Geisler S., Holmström K., Skujat D., Fiesel F., Rothfuss O.C., Kahle P.J., Springer W. (2010). PINK1/Parkin-mediated mitophagy is dependent on VDAC1 and p62/SQSTM1. Nat. Cell Biol..

[64] Ricciardi L., Petrucci S., Guidubaldi A., Ialongo T., Serra L., Ferraris A., Spanò B., Bozzali M., Valente E.M., Bentivoglio A.R. (2014). Phenotypic variability of PINK1 expression: 12 Years’ clinical follow-up of two Italian families. Mov. Disord..

[65] Piredda R., Desmarais P., Masellis M., Gasca-Salas C. (2019). Cognitive and psychiatric symptoms in genetically determined Parkinson’s disease: A systematic review. Eur. J. Neurol..

[66] Nishioka K., Kefi M., Jasinska-Myga B., Wider C., Vilarino-Guell C., Ross O., Heckman M.G., Middleton L.T., Ishihara-Paul L., Gibson R. (2009). A comparative study of LRRK2, PINK1 and genetically undefined familial Parkinson’s disease. J. Neurol. Neurosurg. Psychiatry.

[67] Hedrich K., Hagenah J., Djarmati A., Hiller A., Lohnau T., Lasek K., Grünewald A., Hilker R., Steinlechner S., Boston H. (2006). Clinical Spectrum of Homozygous and Heterozygous PINK1 Mutations in a Large German Family with Parkinson Disease. Arch. Neurol..

[68] Puschmann A., Fiesel F., Caulfield T.R., Hudec R., Ando M., Truban D., Hou X., Ogaki K., Heckman M.G., James E.D. (2017). Heterozygous PINK1 p.G411S increases risk of Parkinson’s disease via a dominant-negative mechanism. Brain.

[69] Krohn L., Grenn F.P., Makarious M.B., Kim J.J., Bandres-Ciga S., Roosen D.A., Gan-Or Z., Nalls M.A., Singleton A.B., Blauwendraat C. (2020). Comprehensive assessment of PINK1 variants in Parkinson’s disease. Neurobiol. Aging.

[70] Schneider S.A., Klein C., Adam M.P., Ardinger H.H., Pagon R.A., Wallace S.E., Bean L.J., Mirzaa G., Amemiya A. (1993). PINK1 Type of Young-Onset Parkinson Disease. GeneReviews^®^.

[71] Samaranch L., Lorenzo-Betancor O., Arbelo J.M., Ferrer I., Lorenzo E., Irigoyen J., Pastor M.A., Marrero C., Isla C., Herrera-Henriquez J. (2010). PINK1-Linked Parkinsonism Is Associated with Lewy Body Pathology. Brain.

[72] Gelmetti V., Ferraris A., Brusa L., Romano F., Lombardi F., Barzaghi C., Stanzione P., Garavaglia B., Dallapiccola B., Valente E.M. (2008). Late Onset Sporadic Parkinson’s Disease Caused by PINK1 Mutations: Clinical and Functional Study: Late Onset Sporadic PD Due to PINK1 Mutations. Mov. Disord..

[73] Ephraty L., Porat O., Israeli D., Cohen O.S., Tunkel O., Yael S., Hatano Y., Hattori N., Hassin-Baer S. (2007). Neuropsychiatric and Cognitive Features in Autosomal-Recessive Early Parkinsonism Due to PINK1 Mutations. Mov. Disord..

[74] Albanese A., Valente E.M., Romito L.M., Bellacchio E., Elia A.E., Dallapiccola B. (2005). The PINK1 Phenotype Can Be Indistinguishable from Idiopathic Parkinson Disease. Neurology.

[75] Savettieri G., Annesi G., Civitelli D., Cirò Candiano I.C., Salemi G., Ragonese P., Annesi F., Tarantino P., Terruso V., D’Amelio M. (2008). Identification of the Novel D297fsX318 PINK1 Mutation and Phenotype Variation in a Family with Early-Onset Parkinson’s Disease. Parkinsonism Relat. Disord..

[76] Doostzadeh J., Tetrud J.W., Allen-Auerbach M., Langston J.W., Schüle B. (2007). Novel Features in a Patient Homozygous for the L347P Mutation in the PINK1 Gene. Parkinsonism Relat. Disord..

[77] Tuin I., Voss U., Kessler K., Krakow K., Hilker R., Morales B., Steinmetz H., Auburger G. (2008). Sleep Quality in a Family with Hereditary Parkinsonism (PARK6). Sleep Med..

[78] Hiller A., Hagenah J.M., Djarmati A., Hedrich K., Reetz K., Schneider-Gold C., Kress W., Münchau A., Klein C. (2007). Phenotypic Spectrum of PINK1-Associated Parkinsonism in 15 Mutation Carriers from 1 Family. Mov. Disord..

[79] Leutenegger A.-L., Salih M.A.M., Ibanez P., Mukhtar M.M., Lesage S., Arabi A., Lohmann E., Durr A., Ahmed A.E.M., Brice A. (2006). Juvenile-Onset Parkinsonism as a Result of the First Mutation in the Adenosine Triphosphate Orientation Domain of PINK1. Arch. Neurol..

[80] Ramirez-Zamora A., Ostrem J.L. (2018). Globus Pallidus Interna or Subthalamic Nucleus Deep Brain Stimulation for Parkinson Disease. JAMA Neurol..

[81] Moro E., Volkmann J., König I., Winkler S., Hiller A., Hassin-Baer S., Herzog J., Schnitzler A., Lohmann K., Pinsker M.O. (2008). Bilateral subthalamic stimulation in Parkin and PINK1 parkinsonism. Neurology.

[82] Borellini L., Cogiamanian F., Carrabba G., Locatelli M., Rampini P., Di Fonzo A., Bana C., Barbieri S., Ardolino G. (2017). Globus pallidus internus deep brain stimulation in PINK-1 related Parkinson’s disease: A case report. Parkinsonism Relat. Disord..

[83] Borellini L., Cogiamanian F., Ardolino G. (2021). Globus pallidus internus deep brain stimulation in PINK-1 related Parkinson’s disease: An update. Parkinsonism Relat. Disord..

[84] Johansen K.K., Jørgensen J.V., White L.R., Farrer M., Aasly J.O. (2011). Parkinson-related genetics in patients treated with deep brain stimulation. Acta Neurol. Scand..

[85] Nakahara K., Ueda M., Yamada K., Koide T., Yoshimochi G., Funayama M., Kim J.-H., Yamakawa S., Mori A., Misumi Y. (2014). Juvenile-onset parkinsonism with digenic parkin and PINK1 mutations treated with subthalamic nucleus stimulation at 45years after disease onset. J. Neurol. Sci..

[86] Matsuda N., Sato S., Shiba K., Okatsu K., Saisho K., Gautier C.A., Sou Y.-S., Saiki S., Kawajiri S., Sato F. (2010). PINK1 stabilized by mitochondrial depolarization recruits Parkin to damaged mitochondria and activates latent Parkin for mitophagy. J. Cell Biol..

[87] Ordureau A., Sarraf S., Duda D.M., Heo J.-M., Jedrychowski M.P., Sviderskiy V., Olszewski J.L., Koerber J.T., Xie T., Beausoleil S.A. (2014). Quantitative Proteomics Reveal a Feedforward Mechanism for Mitochondrial PARKIN Translocation and Ubiquitin Chain Synthesis. Mol. Cell.

[88] Bellin M., Marchetto M.C., Gage F.H., Mummery C. (2012). Induced pluripotent stem cells: The new patient?. Nat. Rev. Mol. Cell Biol..

[89] Imaizumi Y., Okada Y., Akamatsu W., Koike M., Kuzumaki N., Hayakawa H., Nihira T., Kobayashi T., Ohyama M., Sato S. (2012). Mitochondrial dysfunction associated with increased oxidative stress and α-synuclein accumulation in PARK2 iPSC-derived neurons and postmortem brain tissue. Mol. Brain.

[90] Chung S.Y., Kishinevsky S., Mazzulli J.R., Graziotto J., Mrejeru A., Mosharov E.V., Puspita L., Valiulahi P., Sulzer D., Milner T.A. (2016). Parkin and PINK1 Patient iPSC-Derived Midbrain Dopamine Neurons Exhibit Mitochondrial Dysfunction and α-Synuclein Accumulation. Stem Cell Rep..

[91] Key J., Sen N.E., Arsović A., Krämer S., Hülse R., Khan N.N., Meierhofer D., Gispert S., Koepf G., Auburger G. (2020). Systematic Surveys of Iron Homeostasis Mechanisms Reveal Ferritin Superfamily and Nucleotide Surveillance Regulation to be Modified by PINK1 Absence. Cells.

[92] Yamaguchi A., Ishikawa K.-I., Inoshita T., Shiba-Fukushima K., Saiki S., Hatano T., Mori A., Oji Y., Okuzumi A., Li Y. (2020). Identifying Therapeutic Agents for Amelioration of Mitochondrial Clearance Disorder in Neurons of Familial Parkinson Disease. Stem Cell Rep..

[93] Cummins N., Götz J. (2017). Shedding light on mitophagy in neurons: What is the evidence for PINK1/Parkin mitophagy in vivo?. Cell. Mol. Life Sci..

[94] Whitworth A.J., Pallanck L.J. (2017). PINK1/Parkin mitophagy and neurodegeneration—What do we really know in vivo?. Curr. Opin. Genet. Dev..

[95] Fiesel F., Ando M., Hudec R., Hill A.R., Castanedes-Casey M., Caulfield T.R., Moussaud-Lamodière E.L., Stankowski J.N., Bauer P., Betancor O.L. (2015). (Patho-)physiological relevance of PINK 1-dependent ubiquitin phosphorylation. EMBO Rep..

[96] Pickrell A.M., Huang C.-H., Kennedy S.R., Ordureau A., Sideris D.P., Hoekstra J.G., Harper J.W., Youle R.J. (2015). Endogenous Parkin Preserves Dopaminergic Substantia Nigral Neurons following Mitochondrial DNA Mutagenic Stress. Neuron.

[97] Greene J.C., Whitworth A., Kuo I., Andrews L.A., Feany M., Pallanck L.J. (2003). Mitochondrial pathology and apoptotic muscle degeneration in Drosophila parkin mutants. Proc. Natl. Acad. Sci. USA.

[98] Pesah Y., Pham T., Burgess H., Middlebrooks B., Verstreken P., Zhou Y., Harding M., Bellen H., Mardon G. (2004). Drosophila parkinmutants have decreased mass and cell size and increased sensitivity to oxygen radical stress. Development.

[99] Clark I.E., Dodson M.W., Jiang C., Cao J.H., Huh J.R., Seol J.H., Yoo S.J., Hay B.A., Guo M. (2006). Drosophila pink1 is required for mitochondrial function and interacts genetically with parkin. Nat. Cell Biol..

[100] Park J., Lee S.B., Lee S., Kim Y., Song S., Kim S., Bae E., Kim J., Shong M., Kim J.-M. (2006). Mitochondrial dysfunction in Drosophila PINK1 mutants is complemented by parkin. Nat. Cell Biol..

[101] Yang Y., Gehrke S., Imai Y., Huang Z., Ouyang Y., Wang J., Yang L., Beal M.F., Vogel O.H., Lu B. (2006). Mitochondrial pathology and muscle and dopaminergic neuron degeneration caused by inactivation of Drosophila Pink1 is rescued by Parkin. Proc. Natl. Acad. Sci. USA.

[102] Todd A.M., Staveley B.E. (2008). Pink1 suppresses α-synuclein-induced phenotypes in a Drosophila model of Parkinson’s disease. Genome.

[103] De Castro I.P., Costa A.C., Lam D., Tufi R., Fedele V., Moisoi N., Dinsdale D., Deas E., Loh S.H.Y., Martins L.M. (2012). Genetic analysis of mitochondrial protein misfolding in Drosophila melanogaster. Cell Death Differ..

[104] Vincow E.S., Merrihew G., Thomas R.E., Shulman N.J., Beyer R.P., MacCoss M.J., Pallanck L.J. (2013). The PINK1-Parkin pathway promotes both mitophagy and selective respiratory chain turnover in vivo. Proc. Natl. Acad. Sci. USA.

[105] Gandhi S., Wood-Kaczmar A., Yao Z., Plun-Favreau H., Deas E., Klupsch K., Downward J., Latchman D.S., Tabrizi S.J., Wood N.W. (2009). PINK1-Associated Parkinson’s Disease Is Caused by Neuronal Vulnerability to Calcium-Induced Cell Death. Mol. Cell.

[106] Tufi R., Gandhi S., de Castro I.P., Lehmann S., Angelova P.R., Dinsdale D., Deas E., Plun-Favreau H., Nicotera P., Abramov A. (2014). Enhancing nucleotide metabolism protects against mitochondrial dysfunction and neurodegeneration in a PINK1 model of Parkinson’s disease. Nat. Cell Biol..

[107] Lehmann S., Loh S.H.Y., Martins L.M. (2017). Enhancing NAD+ salvage metabolism is neuroprotective in a PINK1 model of Parkinson’s disease. Biol. Open.

[108] Lee K.-S., Huh S., Lee S., Wu Z., Kim A.-K., Kang H.-Y., Lu B. (2018). Altered ER–mitochondria contact impacts mitochondria calcium homeostasis and contributes to neurodegeneration in vivo in disease models. Proc. Natl. Acad. Sci. USA.

[109] Rizzuto R., Brini M., Murgia M., Pozzan T. (1993). Microdomains with High Ca^2+^ Close to IP 3-Sensitive Channels that Are Sensed by Neighboring Mitochondria. Science.

[110] Lee S., Lee K.-S., Huh S., Liu S., Lee D.-Y., Hong S.H., Yu K., Lu B. (2016). Polo Kinase Phosphorylates Miro to Control ER-Mitochondria Contact Sites and Mitochondrial Ca^2+^ Homeostasis in Neural Stem Cell Development. Dev. Cell.

[111] Sheng Z.-H., Cai Q. (2012). Mitochondrial transport in neurons: Impact on synaptic homeostasis and neurodegeneration. Nat. Rev. Neurosci..

[112] Wang X., Winter D., Ashrafi G., Schlehe J., Wong Y.L., Selkoe D., Rice S., Steen J., LaVoie M., Schwarz T.L. (2011). PINK1 and Parkin Target Miro for Phosphorylation and Degradation to Arrest Mitochondrial Motility. Cell.

[113] Liu S., Sawada T., Lee S., Yu W., Silverio G., Alapatt P., Millan I., Shen A., Saxton W., Kanao T. (2012). Parkinson’s Disease–Associated Kinase PINK1 Regulates Miro Protein Level and Axonal Transport of Mitochondria. PLoS Genet..

[114] Lee Y., Stevens D.A., Kang S.-U., Jiang H., Lee Y.-I., Ko H.S., Scarffe L.A., Umanah G.E., Kang H., Ham S. (2017). PINK1 Primes Parkin-Mediated Ubiquitination of PARIS in Dopaminergic Neuronal Survival. Cell Rep..

[115] Shin J.-H., Ko H.S., Kang H., Lee Y., Lee Y.-I., Pletinkova O., Troconso J.C., Dawson V.L., Dawson T.M. (2011). PARIS (ZNF746) Repression of PGC-1α Contributes to Neurodegeneration in Parkinson’s Disease. Cell.

[116] Pirooznia S.K., Yuan C., Khan M.R., Karuppagounder S.S., Wang L., Xiong Y., Kang S.U., Lee Y., Dawson V.L., Dawson T.M. (2020). PARIS induced defects in mitochondrial biogenesis drive dopamine neuron loss under conditions of parkin or PINK1 deficiency. Mol. Neurodegener..

[117] Decout A., Katz J.D., Venkatraman S., Ablasser A. (2021). The cGAS–STING pathway as a therapeutic target in inflammatory diseases. Nat. Rev. Immunol..

[118] Sliter D.A., Martinez J., Hao L., Chen X., Sun N., Fischer T.D., Burman J.L., Li Y., Zhang Z., Narendra D.P. (2018). Parkin and PINK1 mitigate STING-induced inflammation. Nat. Cell Biol..

[119] Lee J.J., Andreazza S., Whitworth A.J. (2020). The STING pathway does not contribute to behavioural or mitochondrial phenotypes in Drosophila Pink1/parkin or mtDNA mutator models. Sci. Rep..

[120] Julienne H., Buhl E., Leslie D.S., Hodge J.J. (2017). Drosophila PINK1 and parkin loss-of-function mutants display a range of non-motor Parkinson’s disease phenotypes. Neurobiol. Dis..

[121] Cornelissen T., Vilain S., Vints K., Gounko N., Verstreken P., Vandenberghe W. (2018). Deficiency of parkin and PINK1 impairs age-dependent mitophagy in Drosophila. eLife.

[122] Han H., Tan J., Wang R., Wan H., He Y., Yan X., Guo J., Gao Q., Li J., Shang S. (2020). PINK 1 phosphorylates Drp1 S616 to regulate mitophagy-independent mitochondrial dynamics. EMBO Rep..

[123] Terriente-Felix A., Wilson E.L., Whitworth A.J. (2020). Drosophila phosphatidylinositol-4 kinase fwd promotes mitochondrial fission and can suppress Pink1/parkin phenotypes. PLoS Genet..

[124] Han Y., Zhuang N., Wang T. (2021). Roles of PINK1 in regulation of systemic growth inhibition induced by mutations of PTEN in Drosophila. Cell Rep..

[125] Lazarou M., Sliter D.A., Kane L.A., Sarraf S.A., Wang C., Burman J.L., Sideris D.P., Fogel A.I., Youle R.J. (2015). The ubiquitin kinase PINK1 recruits autophagy receptors to induce mitophagy. Nature.

[126] Wang H.-L., Chou A.-H., Wu A.-S., Chen S.-Y., Weng Y.-H., Kao Y.-C., Yeh T.-H., Chu P.-J., Lu C.-S. (2011). PARK6 PINK1 mutants are defective in maintaining mitochondrial membrane potential and inhibiting ROS formation of substantia nigra dopaminergic neurons. Biochim. Biophys. Acta Mol. Basis Dis..

[127] Wang H.-L., Chou A.-H., Yeh T.-H., Li A.H., Chen Y.-L., Kuo Y.-L., Tsai S.-R., Yu S.-T. (2007). PINK1 mutants associated with recessive Parkinson’s disease are defective in inhibiting mitochondrial release of cytochrome c. Neurobiol. Dis..

[128] Gautier C.A., Kitada T., Shen J. (2008). Loss of PINK1 causes mitochondrial functional defects and increased sensitivity to oxidative stress. Proc. Natl. Acad. Sci. USA.

[129] McWilliams T., Prescott A.R., Allen G.F., Tamjar J., Munson M.J., Thomson C., Muqit M.M., Ganley I.G. (2016). mito-QC illuminates mitophagy and mitochondrial architecture in vivo. J. Cell Biol..

[130] Sun N., Yun J., Liu J., Malide D., Liu C., Rovira I.I., Holmström K., Fergusson M.M., Yoo Y.H., Combs C.A. (2015). Measuring In Vivo Mitophagy. Mol. Cell.

[131] Stevens D.A., Lee Y., Kang H.C., Lee B.D., Lee Y.-I., Bower A., Jiang H., Kang S.-U., Andrabi S.A., Dawson V.L. (2015). Parkin loss leads to PARIS-dependent declines in mitochondrial mass and respiration. Proc. Natl. Acad. Sci. USA.

[132] Trifunovic A., Wredenberg A., Falkenberg M., Spelbrink J., Rovio A.T., Bruder C.E., Bohlooly-Y M., Gidlöf S., Oldfors A., Wibom R. (2004). Premature ageing in mice expressing defective mitochondrial DNA polymerase. Nat. Cell Biol..

[133] Hang L., Thundyil J., Goh G.W.Y., Lim K.-L. (2019). AMP Kinase Activation is Selectively Disrupted in the Ventral Midbrain of Mice Deficient in Parkin or PINK1 Expression. Neuromol. Med..

[134] Gispert S., Brehm N., Weil J., Seidel K., Rüb U., Kern B., Walter M., Roeper J., Auburger G. (2014). Potentiation of neurotoxicity in double-mutant mice with Pink1 ablation and A53T-SNCA overexpression. Hum. Mol. Genet..

[135] McWilliams T.G., Barini E., Pohjolan-Pirhonen R., Brooks S.P., Singh F., Burel S., Balk K., Kumar A., Garriga L.M., Prescott A.R. (2018). Phosphorylation of Parkin at serine 65 is essential for its activation in vivo. Open Biol..

[136] Zhi L., Qin Q., Muqeem T., Seifert E.L., Liu W., Zheng S., Li C., Zhang H. (2019). Loss of PINK1 causes age-dependent decrease of dopamine release and mitochondrial dysfunction. Neurobiol. Aging.

[137] Sardi S.P., Cedarbaum J.M., Brundin P. (2018). Targeted Therapies for Parkinson’s Disease: From Genetics to the Clinic. Mov. Disord..

[138] Palikaras K., Daskalaki I., Markaki M., Tavernarakis N. (2017). Mitophagy and age-related pathologies: Development of new therapeutics by targeting mitochondrial turnover. Pharmacol. Ther..

[139] Georgakopoulos N.D., Wells G., Campanella N.D.G.M. (2017). The pharmacological regulation of cellular mitophagy. Nat. Chem. Biol..

[140] Liu J., Liu W., Li R., Yang H. (2019). Mitophagy in Parkinson’s Disease: From Pathogenesis to Treatment. Cells.

[141] Moors T.E., Hoozemans J.J.M., Ingrassia A., Beccari T., Parnetti L., Chartier-Harlin M.-C., Van De Berg W.D.J. (2017). Therapeutic potential of autophagy-enhancing agents in Parkinson’s disease. Mol. Neurodegener..

[142] East D.A., Fagiani F., Crosby J., Georgakopoulos N.D., Bertrand H., Schaap M., Fowkes A., Wells G., Campanella M. (2014). PMI: A ΔΨm Independent Pharmacological Regulator of Mitophagy. Chem. Biol..

[143] Koentjoro B., Park J.-S., Sue C.M. (2017). Nix restores mitophagy and mitochondrial function to protect against PINK1/Parkin-related Parkinson’s disease. Sci. Rep..

[144] Abudu Y.P., Pankiv S., Mathai B.J., Lamark T., Johansen T., Simonsen A. (2019). NIPSNAP1 and NIPSNAP2 act as “eat me” signals to allow sustained recruitment of autophagy receptors during mitophagy. Autophagy.

[145] Bingol B., Tea J., Phu L., Reichelt M., Bakalarski C., Song Q., Foreman O., Kirkpatrick D., Sheng M. (2014). The mitochondrial deubiquitinase USP30 opposes parkin-mediated mitophagy. Nat. Cell Biol..

[146] Wang Y., Serricchio M., Jauregui M., Shanbhag R., Stoltz T., Di Paolo C.T., Kim P.K., McQuibban G.A. (2015). Deubiquitinating enzymes regulate PARK2-mediated mitophagy. Autophagy.

[147] Cornelissen T., Haddad D., Wauters F., Van Humbeeck C., Mandemakers W., Koentjoro B., Sue C., Gevaert K., De Strooper B., Verstreken P. (2014). The deubiquitinase USP15 antagonizes Parkin-mediated mitochondrial ubiquitination and mitophagy. Hum. Mol. Genet..

[148] Durcan T.M., Tang M.Y., Pérusse J.R., Dashti E.A., Aguileta M.A., McLelland G.-L., Gros P., Shaler T.A., Faubert D., Coulombe B. (2014). USP 8 regulates mitophagy by removing K 6-linked ubiquitin conjugates from parkin. EMBO J..

[149] Miller S., Muqit M.M. (2019). Therapeutic approaches to enhance PINK1/Parkin mediated mitophagy for the treatment of Parkinson’s disease. Neurosci. Lett..

